# Integrating porphyrin-based nanoporous organic polymers with electrochemical aptasensors for ultratrace detection of kanamycin

**DOI:** 10.1007/s00604-024-06180-z

**Published:** 2024-01-17

**Authors:** Guanghui Tian, Feng Guo, Chuanbin Fan, Zi-Ao Zong, Junli Wang, Zhuorigebatu Tegudeer, Wen-Yang Gao

**Affiliations:** 1 School of Laboratory Medicine, Youjiang Medical University for Nationalities, Baise, 533000 Guangxi China; 2 Department of Chemistry and Biochemistry, Ohio University, Athens, OH 45701 USA

**Keywords:** Porous organic polymers, Electrochemical aptasensors, Electrochemical impedance spectroscopy, Ultratrace detection, Kanamycin

## Abstract

The synthesis and characterization of two new porphyrin-based porous organic polymers (POPs) via Sonogashira cross-coupling reaction and leverage the two obtained POPs is reported for the fabrication of electrochemical aptasensors to detect kanamycin at an ultratrace level. The resultant electrochemical aptasensor demonstrates a high linear relationship with the logarithmic value of kanamycin concentration in the range 5 × 10^−5^–5 μg/L with the limit of detection of 17.6 pg/L or 36.3 fM. During the analysis of real samples from milk and river, a relative standard deviation of less than 4.39%, and good recovery values in the range 97.0–105% were obtained.

**Graphical Abstract:**

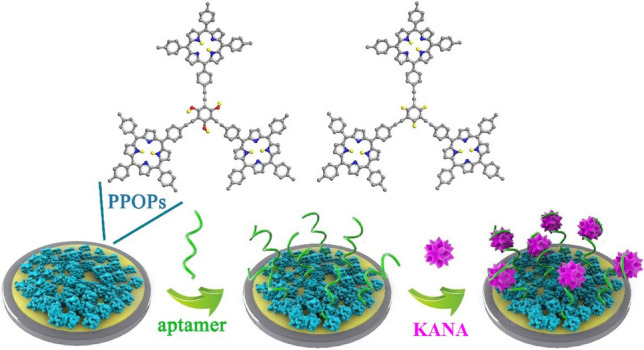

**Supplementary Information:**

The online version contains supplementary material available at 10.1007/s00604-024-06180-z.

## Introduction

Strategies to track down environmental pathways of antibiotics are becoming urgently needed, as their residues being found in underground water, river, soils, and agricultural by-products raise alarming concerns to the ecological system and human health [1, 2]. For instance, kanamycin (KANA), one of the commonly used antibiotics to treat severe bacterial infections and tuberculosis, has left its footprint in a variety of environmental domains. Current methods to detect KANA include enzyme-linked immunosorbent assay [3], high-performance liquid chromatography coupled with various detectors [4], photoelectrochemistry [5], and surface-enhanced Raman spectroscopy [6]. However, these analytical approaches suffer from drawbacks of high cost, limited sensitivity, tedious preprocessing, and complicated time-consuming operations. Thus, an effective and efficient detection tool for KANA is still highly desirable.

Meanwhile, electrochemical aptasensors, a type of biosensors, leverage aptamers as recognition elements and monitor changes of certain electrical signal (i.e., current, impedance, and potential) in response to a recognition event [7]. Due to cooperative behaviors of aptamer binding specificity and the sensitivity of low-cost and fast electrochemical techniques [8–13], electrochemical aptasensors are emerging into an alternative yet promising tool to keep track of antibiotics in the environment.

While several types of materials including graphene, metal–organic frameworks, and gold nanoparticles [14–20] have been employed to immobilize aptamers and amplify the electrochemical output signal, the ongoing development of functional porous organic polymers (POPs) [21, 22], composed of exclusively organic components connected via covalent bonds, leads themselves to be a potential family of candidates to host aptamers [23–26] for the trace detection of KANA, given their remarkable chemical stability, high porosity, and precise synthetic tunability. Herein, we report our systemic work on assembling electrochemical aptasensors using porphyrin-based POPs (PPOPs) for monitoring KANA in real samples at the ultra-trace level. The rationale to exploit the porphyrin as the strut unit is to facilitate the formation of rigid and significant pore space and the immobilization of aptamers via intermolecular π–π stacking.

### Experimental

**Fig. 1:**
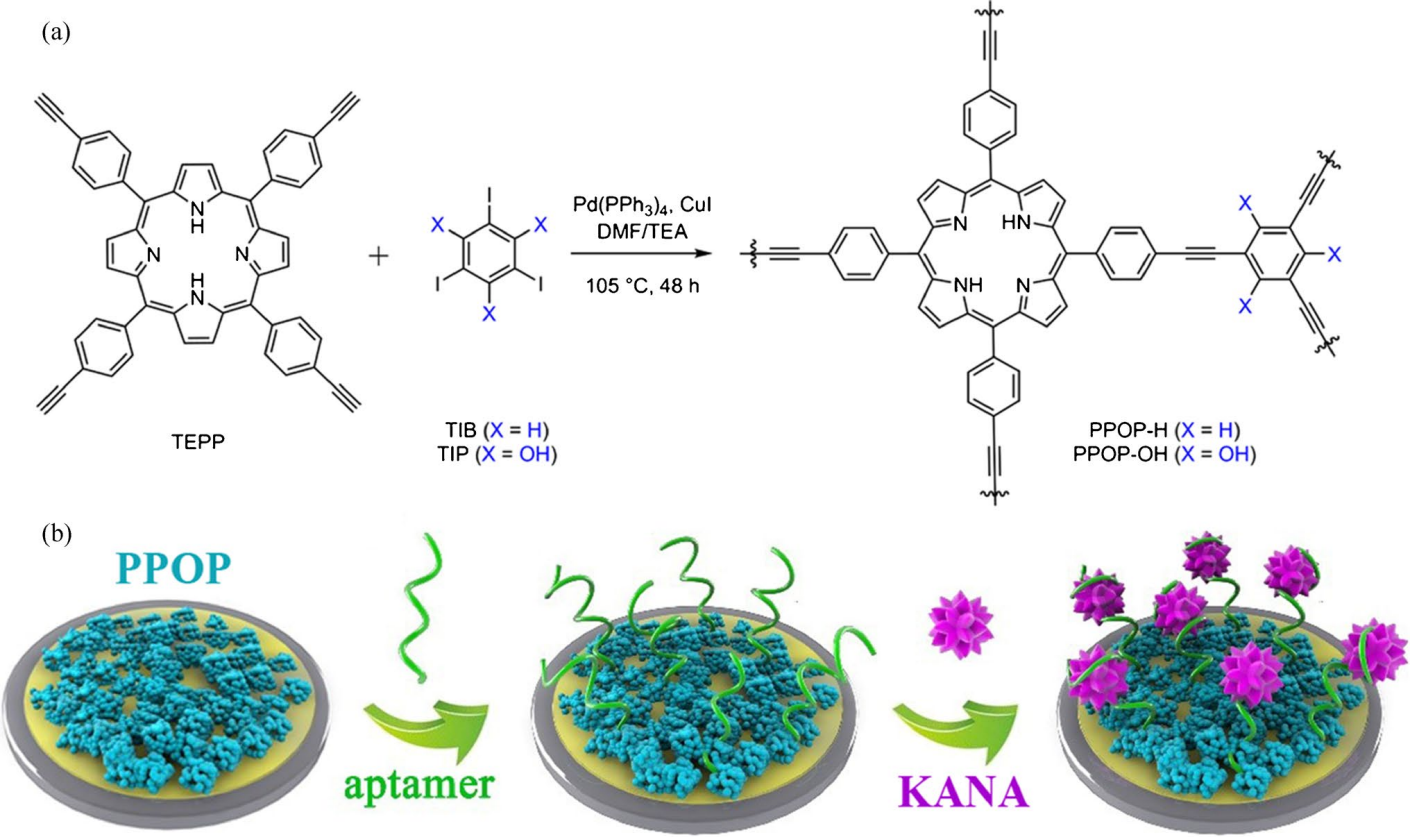
**a** Two porphyrin-based porous organic polymers were prepared by Sonogashira cross-coupling chemistry. **b** An illustration of fabricating PPOP-based electrochemical aptasensors for detecting KANA

We started the assembly of electrochemical aptasensors by synthesizing two porphyrin-based POPs via Sonogashira cross-coupling chemistry between the porphyrin-containing polyalkyne and aryl iodides (Fig. 1a). In particular, the coupling reaction was carried out using a mixture of 5,10,15,20-tetrakis(4-ethynylphenyl)-21*H*,23*H*-porphine (TEPP, 0.75 equiv) with 1,3,5-triiodobenzene (TIB, 1.0 equiv) or 2,4,6-triiodophloroglucinol (TIP, 1.0 equiv) in 1:1 (v/v) *N,N*-dimethylformamide (DMF) and triethylamine, which was heated at 105 °C for 48 h under a N_2_ atmosphere (see details in Supplementary Information, SI). The harvested purple powder solids (Figure S1), PPOP-H from the monomer of TIB and PPOP-OH from TIP, did not dissolve in any common solvents, such as DMF, tetrahydrofuran, methanol, or H_2_O. Extensive washing procedures were applied to the obtained solids using the above solvents, as well as 0.1 M aqueous HCl solution, to remove potential residues. The obtained PPOPs serve as potential candidates to immobilize aptamers and thus lead to electrochemical aptasensors for sensing KANA (Fig. 1b).

## Results and discussion

**Fig. 2:**
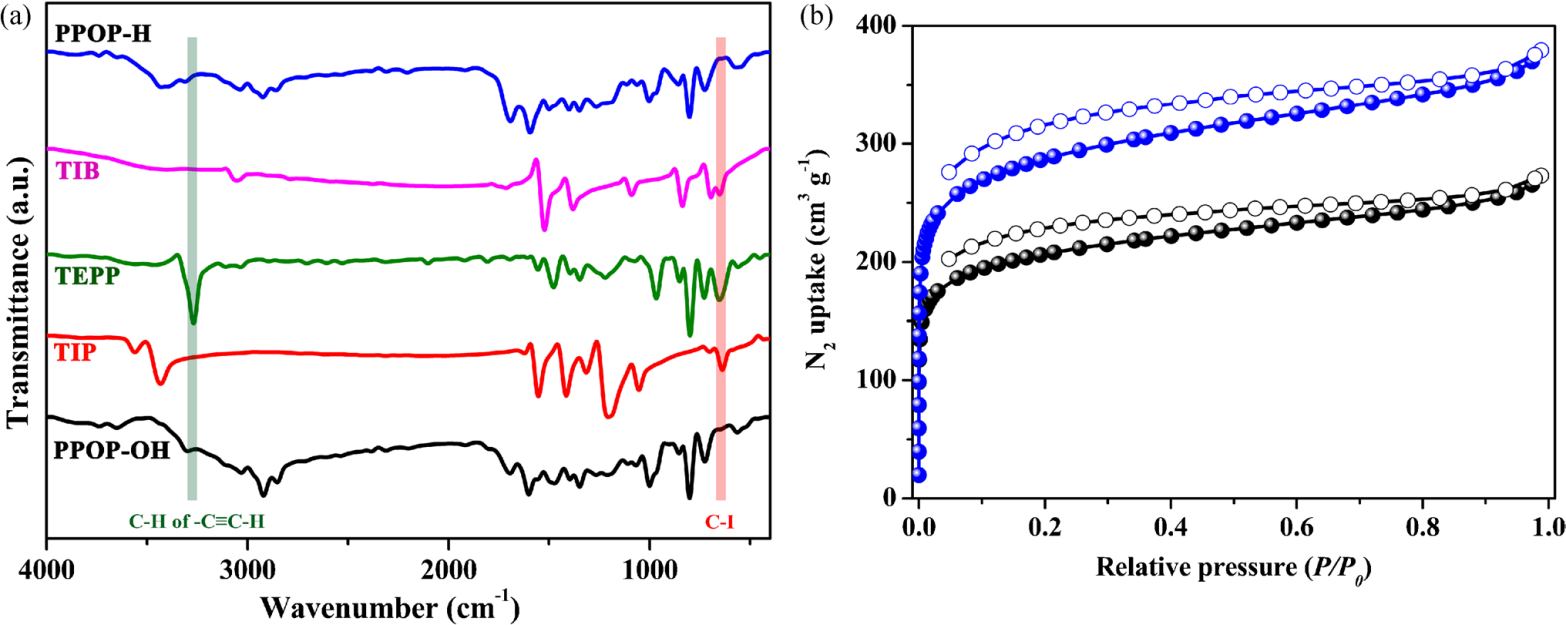
**a** IR spectra indicate disappearances of terminal alkyne C–H vibration around 3270 cm^−1^ from TEPP and C–I stretch around 600 cm^−^.^1^ from TIB and TIP in the obtained polymers. **b** N_2_ adsorption isotherms at 77 K were collected on samples of PPOP-H (blue line) and PPOP-OH (black line)

The successful high-degree polymerization reactions were monitored by both solid-state ^13^C cross-polarization magic angle spinning (CP MAC) nuclear magnetic resonance (NMR) and infrared (IR) spectroscopy. As shown in Figure S2, both PPOP-H and PPOP-OH display signals between 110 and 154 ppm corresponding to aromatic carbons from porphyrin and phenyl motifs. The relatively broad peak around 76 ppm is ascribed to –C≡C– fragment in the skeleton. IR spectra (Fig. 2a) indicate disappearances of terminal alkyne C–H vibration around 3270 cm^−1^ from TEPP and C–I stretch around 600 cm^−1^ from TIB and TIP in the obtained polymers. On the basis of power X-ray diffraction analysis (Figures S3 and S4), PPOP-OH and PPOP-H are amorphous materials in the absence of any diffraction peaks, which is consistent with previous reports that Sonogashira cross-coupling reactions provide access to amorphous POPs due to the irreversibility nature of their reactions [27, 28]. Thermogravimetric analysis (TGA, Figures S5 and S6) reveals that both POPs are thermally stable to above 320 °C after which continuous weight loss is observed.

To investigate their porosity, N_2_ adsorption isotherms at 77 K (Fig. 2b) were collected on samples of PPOP-H and PPOP-OH, which were pre-exchanged by dichloromethane and further activated under vacuum at 120 °C for 10 h. Brunauer–Emmet–Teller (BET) surface areas are calculated as 701 m^2^/g and 976 m^2^/g (P/P_0_ = 0.05–0.25) for PPOP-OH and PPOP-H, respectively. Furthermore, their pore size distributions in Figure S7 derived from N_2_ adsorption isotherms show both samples feature hierarchical pores. Scanning electron microscopy (SEM) and transmission electron microscopy (TEM) were also employed to observe the morphologies and microstructures, which show that obtained PPOPs are crosslinked amorphous nanoparticles (Figure S8).

Given the features of high stability and hierarchical pores along with extended conjugated motifs, the obtained two PPOPs were readily used to modify the surface of Au electrode (AE) to obtain PPOP@AE, which were then immersed in the aptamer solution to build the titled PPOP-based electrochemical aptasensors (apt@PPOP@AE, see details in SI). Electrochemical impedance spectroscopy (EIS) Nyquist plots were simulated by the Randles equivalent circuit (Figure S9) [29]. The *R*_ct_ changes (Δ*R*_ct_ = *R*_ct,PPOP@AE_ − *R*_ct,AE_ and Δ*R*_ct_ = *R*_ct,apt@PPOP@AE_ − *R*_ct,PPOP@AE_) were calculated from a series of screening experiments to obtain optimal preparation conditions. For instance, in the case of apt@PPOP-OH@AE, different amounts of PPOP-OH were loaded on the surface of AEs. As shown in Figure S10, the Δ*R*_ct_ value of PPOP-OH@AE increases to 60 Ω after adding 10 μL of PPOP-OH (0.20 mg/mL in methanol), but no significant increase can be found with the further addition of PPOP-OH. Additionally, the Δ*R*_ct_ value of apt@PPOP-OH@AE (Figure S11) was significantly enhanced by extending its soaking time in the aptamer solution at 10.0 ng/mL for 2 h. Thus, those results offer the optimal preparation condition to prepare the PPOP-OH-based electrochemical aptasensor.

To better understand the fabricated PPOP-OH-based electrochemical aptasensor, a suite of solid-state characterizations was applied to the composite samples, apt@PPOP-OH or apt@PPOP-OH@AE. High-angle annular dark field-scanning transmission electron microscopy (HAADF-STEM) coupled with energy-dispersive X-ray spectroscopy (EDX) shows that C and N elements are well dispersed on apt@PPOP-OH (Figures S12a–c). Meanwhile, the P element from aptamer is also uniformly dispersed throughout the sample (Figure S12d), which confirms the successful fabrication of apt@PPOP-OH via immersion. X-ray photoelectron spectroscopy (XPS) was employed to probe the carbon electronic states of PPOP-H and apt@PPOP-H. As seen in Figure S12e, the high-resolution C1*s* XPS spectrum of PPOP-OH can be well deconvoluted into three peaks at 284.4 eV (C–C/C–H), 285.5 eV (C–O), and 287.4 eV (C–N = C). Nevertheless, four separate peaks are found in apt@PPOP-OH, including 284.4 eV (C–C/C–H), 285.5 eV (C–O), 287.3 eV (C–N = C), and 291.1 eV (π–π) (Figure S12f). The additional peak at 291.1 eV indicates that aptamers are immobilized on PPOP-OH via the π–π interaction, which is consistent with the previous report [30].

**Fig. 3:**
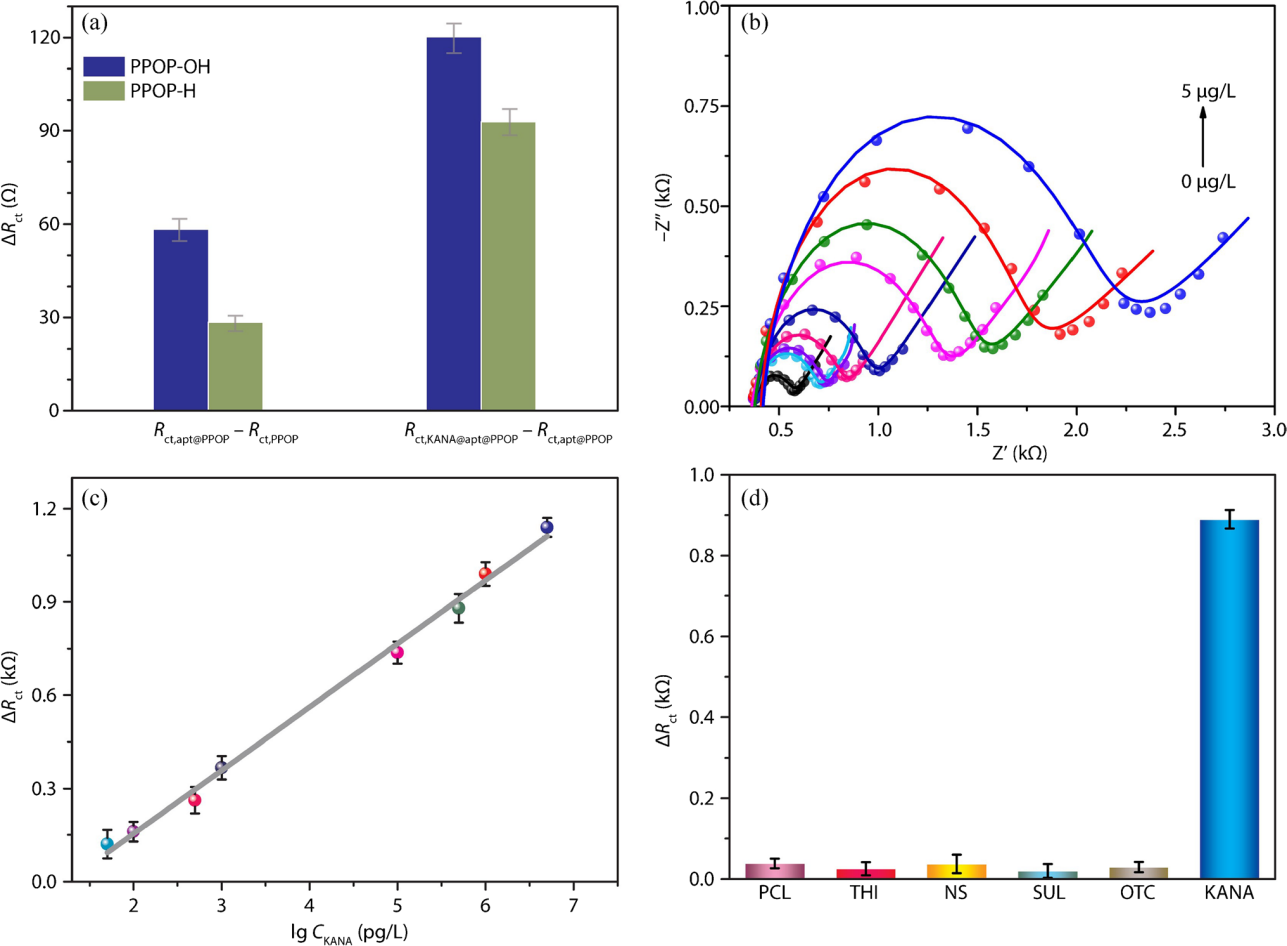
**a** Comparison of the Δ*R*_ct_ values of PPOP-OH and PPOP-H-based aptasensors indicates the relatively high sensitivity using PPOP-OH. **b** EIS Nyquist plots of the PPOP-OH-based electrochemical aptasensor in different KANA concentrations. **c** The linear relationship between the Δ*R*_ct_ value and the logarithmic value of KANA concentration. **d** The selectivity of the fabricated PPOP-OH-based electrochemical aptasensor for detecting KANA in the presence of various interferences at 0.5 μg/L

The obtained electrochemical aptasensor, apt@PPOP-OH@AE, was employed to probe KANA. EIS Nyquist plots were used to monitor the electrochemical signal change during the manufacturing and testing processes. As shown in Figure S13, apt@PPOP-OH@AE displays larger EIS Nyquist plots than PPOP-OH@AE (Δ*R*_ct_ = *R*_ct,apt@PPOP-OH@AE_ − *R*_ct,PPOP-OH@AE_ = 58.4 Ω). Moreover, the KANA (50 pg/L or 0.05 ppt) treated apt@PPOP-OH@AE sample shows expanded EIS Nyquist plots relative to apt@PPOP-OH@AE itself (Δ*R*_ct_ = *R*_ct,KANA@apt@PPOP-OH@AE_ − *R*_ct, apt@PPOP-OH@AE_ = 120.2 Ω). These data unambiguously highlight that the PPOP-OH-based electrochemical aptasensor is successfully applied to detect KANA. Furthermore, the interaction with KANA was reflected on the C1*s* XPS signal of KANA-loaded apt@PPOP-OH (Figure S12g), which is deconvoluted into four peaks of 284.4 eV (C–C/C–H), 285.9 eV (C–O/C–N), 288.1 eV (C–N = C/C = O), and 291.1 eV (π–π). Compared with apt@PPOP-OH, the slight shifts of peaks are probably attributed to the addition of KANA. Based on the same fabrication procedures, we prepared another electrochemical aptasensor using PPOP-H, apt@PPOP-H@AE. EIS Nyquist plots of the PPOP-H-based electrochemical aptasensor (Figure S14) show performances similar to those of the PPOP-OH-based aptasensor. However, the corresponding Δ*R*_ct_ values (Fig. 3a) of the PPOP-H-based aptasensor are significantly lower than ones of the PPOP-OH-based aptasensor, which is mainly attributed to the relatively strong interaction between aptamer and the –OH functional group in PPOP-OH. Thus, the following studies were carried out by focusing on apt@PPOP-OH@AE for the detection of KANA.

The electrochemical aptasensor, apt@PPOP-OH@AE, was incubated separately in a series of KANA solutions at different concentrations to evaluate its sensitivity. As seen in Fig. 3b, the EIS Nyquist plots were enhanced appreciably and stepwisely upon increasing the KANA concentration, which generated a G-quadruplex complex between KANA and aptamer. The *R*_ct_ change (Δ*R*_ct_ = *R*_ct,KANA@apt@PPOP-OH@AE_ − *R*_ct,apt@PPOP-OH@AE_) was used as the responsive signal to detect KANA. This Δ*R*_ct_ value exhibits a great linear relationship with the logarithmic value of KANA concentration in the range of 5 × 10^−5^–5 μg/L (or ppb). The corresponding linear regression equation is Δ*R*_ct_ = 0.204(± 0.006) × lg *C*_KANA_ − 0.25(± 0.02) with the determination coefficient (*R*^2^) of 0.995 (Fig. 3c). The limit of detection (LOD) is calculated to be 17.6 pg/L or 36.3 fM with a signal-to-noise ratio of 3 through the simulated linear regression equation, which is significantly superior than other reported sensors (see the summary in Table 1) [31–38].

**Table 1:** The obtained electrochemical aptasensor, apt@PPOP-OH@AE, demonstrates superior sensing performance for KANA than other testing methods

Materials	Test method	Detection range	LOD	Ref
Au/SD-TiO_2_ NT	Amperometric	0.2–200 nM	0.1 nM	[31]
GO/w-*g*-C_3_N_4_	Photocurrent	1–230 nM	0.2 nM	[32]
Conducting polymer/Au nanocomposite	Linear sweep voltammetry	0.05–9 μM	9.4 nM	[33]
Imprinted boronic acid-functionalized Au NP composite	Surface plasmon resonance	1–1000 pM	1 pM	[34]
GO/Au NP nanocomposite	Surface-enhanced Raman scattering	1–100 nM	0.75 nM	[35]
Molecularly imprinted polymer	Surface plasmon resonance	0.1–10 μM	12 nM	[36]
TiO_2_-MoS_2_-Au NP heterostructure	Photoelectrochemical aptasensor	0.2–450 μM	50 pM	[37]
Carbon black-oligolactide composite	Electrochemical impedance spectroscopy	0.7–50 nM	0.3 nM	[38]
PPOP-OH	Electrochemical impedance spectroscopy	1.03 × 10^−4^–10.3 nM	36.3 fM	This work

The reproducibility of apt@PPOP-OH@AE was confirmed by four parallel electrodes for impedimetric aptasensing of KANA at 0.5 μg/L, which have similar Δ*R*_ct_ values with a relative standard deviation (RSD) below 4.7% (Figure S15). Moreover, the selectivity of apt@PPOP-OH@AE was studied by analyzing the Δ*R*_ct_ values of the apt@PPOP-OH@AE in KANA and various interferences (Fig. 3d), including penicillin (PCL), thiamphenicol (THI), neomycin sulfate (NS), sulfapyridine (SUL), and oxytetracycline (OTC). The electrochemical aptasensor shows the maximum Δ*R*_ct_ value for detecting KANA (0.5 μg/L), while the negligible variation was found for these interferences at the same concentration (0.5 μg/L), which reveals its excellent selectivity toward KANA. The fabricated aptasensor after monitoring KANA (0.5 μg/L) can be retained well at − 20 °C for 10 days to ascertain the acceptable stability, as displayed in Figure S16. Hence, the PPOP-OH-based electrochemical aptasensor has high selectivity, fine reproducibility, and acceptable stability in the detection of KANA.

Detection of KANA in real samples is an important step to assess the practical applicability of sensors. Here we employed samples of milk and river to investigate the detection capability of apt@PPOP-OH@AE. The electrochemical aptasensor was immersed in real samples together with different amounts of KANA to collect EIS Nyquist plots (see details in S1). As summarized in Table S1, the measured number of KANA is consistent well with the actual KANA addition at 0.100, 1.00, and 100 ng/L with a low RSD (< 4.39%) and a recovery value in the range of 97.0–105%. Therefore, the PPOP-OH-based electrochemical aptasensor illustrates its applicability to detect KANA in various real samples. However, the spent electrochemical aptasensor is a little difficult to restore the original sensing ability by immersing in the distilled water, which may be complicated various factors including strong interactions between aptamers and KANA and KANA being trapped in POPs.

## Conclusion

In conclusion, we not only reported the synthesis and characterization of two porphyrin-based porous organic polymers, but also employed them to build electrochemical aptasensors to detect KANA. Thanks to the existence of hydroxyl functional groups as well as hierarchical pores and extended conjugated motifs, the PPOP-OH-based electrochemical aptasensor shows remarkable impedimetric sensing for ultratrace KANA even in various real samples. The fabricated aptasensor has ultrahigh sensitivity, outstanding selectivity, available reproducibility, and impressive stability. We expect that this research paves a promising avenue to develop highly sensitive electrochemical aptasensors toward ultratrace analytes.

### Supplementary Information

Below is the link to the electronic supplementary material.

Supplementary file1 (DOCX 30472 KB)

## Data Availability

All data generated or analysed during this study are included in this published article and its supplementary information file.
