# Supplementary material for: Integrating porphyrin-based nanoporous organic polymers with electrochemical aptasensors for ultratrace detection of kanamycin
Source: Mikrochim Acta. 2024 Jan 17;191(2):100. doi: 10.1007/s00604-024-06180-z (PMC10794321; doi:10.1007/s00604-024-06180-z)
Supplement: Supplementary file 1 — Supplementary file1 (DOCX 30472 KB) [file 604_2024_6180_MOESM1_ESM.docx]

Supporting Information

**Integrating Porphyrin-based Nanoporous Organic Polymers with Electrochemical Aptasensors for Ultratrace Detection of Kanamycin**

Guanghui Tian,^a^ Feng Guo,*^a^ Chuanbin Fan,^a^ Zi-Ao Zong,^a^ Junli Wang,^a^ Zhuorigebatu Tegudeer,^b^ and Wen-Yang Gao*^b^

a. School of Laboratory Medicine, Youjiang Medical University for Nationalities, Baise, 533000 Guangxi, China. Email: guofeng1510@yeah.net

b. Department of Chemistry and Biochemistry, Ohio University, Athens, Ohio 45701, USA. Email: gaow@ohio.edu

**Table of Contents**

A. General Considerations S2

B. Synthesis and Characterization S2

C. Supporting Data S4

1. **General Considerations**

**Materials** Solvents and chemicals were obtained from companies and used as received, including 5,10,15,20-tetrakis(4-ethynylphenyl)-21*H*,23*H*-porphine (97%, Aladdin), 1,3,5-triiodobenzne (98%, Aladdin), 2,4,6-triiodophloroglucinol (99%, Shengkailun), *N,N*-dimethylformamide (>99.9%, Meryer), triethylamine (>99.5%, Meryer), tetrahydrofuran (99.0%, Meryer), methanol (99.9%, Meryer), tetrakis(triphenylphosphine)palladium(0) (99%, J&K Scientific), copper(I) iodide (CuI, 98%, J&K Scientific), Na_2_HPO_4_ (99%, J&K Scientific), KH_2_PO_4_ (99%, J&K Scientific), KCl (99.5%, Energy Chemical), NaCl (≥ 99.5%, Energy Chemical). The KANA aptamer was purchased from Shanghai Sangon Biotechnology Co., Ltd. The sequence is 5'-TGG GGG TTG AGG CTA AGC CGA-3'. The Au electrode (diameter = 3.0 mm) was purchased from Tianjin Aida Hengsheng Technology Development Co., Ltd.

**Characterization Details** Powder X-ray diffraction (PXRD) patterns were measured on Rigaku SmartLab. Fourier transform infrared (FT-IR) spectra were collected on Bruker ALPHA-T spectrometer. X-ray photoelectron spectra (XPS) spectra were performed on AXIS Ultra DLD X-ray photoelectron spectroscopy. Morphology and microstructure were investigated using a Nova Nano SEM 230 scanning electron microscope (SEM) and Tecnai G^2^ F20 S-TWIN transmission electron microscope (TEM). The ^13^C solid state NMR spectra were recorded on Varian Infinity-plus 400 MHz spectrometer. N_2_ adsorption isotherms were measured at 77 K using a Micromeritics ASAP 2020 surface area analyzer.

**Gas Sorption Details** N_2_ adsorption isotherms (0−1.0 bar pressure range) were measured volumetrically at 77 K using a Micromeritics ASAP 2020 surface area analyzer. The synthesized samples underwent solvent exchange with dichloromethane for 3 d (15 mL × 9). The solvent-exchanged samples were transferred to pre-weighed analysis tubes. The sample was evacuated at 120 °C until the outgas rate was <10 μbar/min and further maintained for 10 h. The tube was weighed to determine the mass of the activated sample. The tube was transferred to the analysis port of the instrument. UHP-grade (99.999% purity) N_2_ and He were used for all adsorption measurements.

1. **Synthesis and Characterization**

**Synthesis of of PPOP-H**


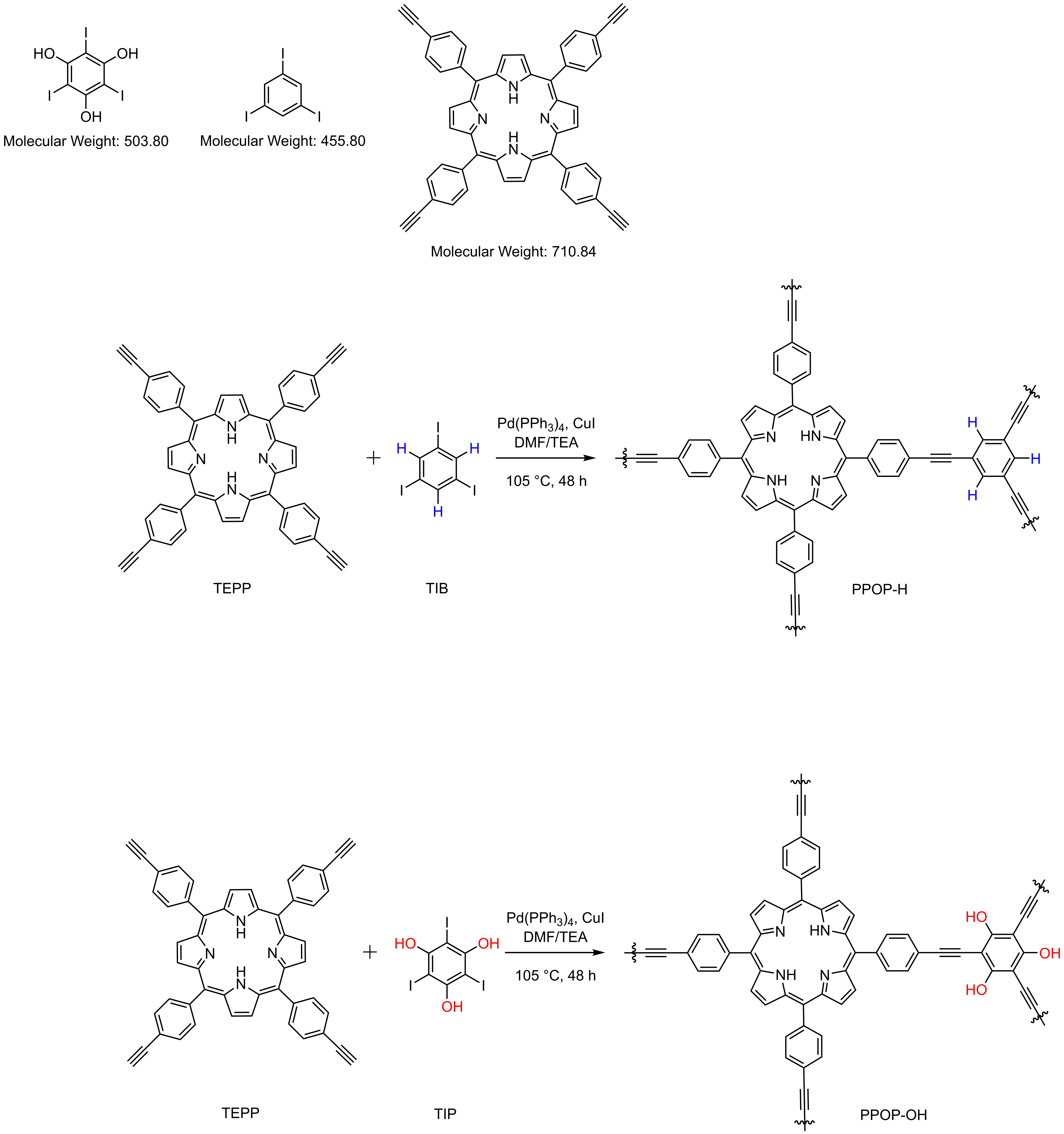


A mixture of TEPP (256 mg, 0.360 mmol, 0.750 equiv), TIB (219 mg, 0.480 mmol, 1.00 equiv), tetrakis(triphenylphosphine)palladium(0) (36 mg, 0.031 mmol, 0.065 equiv), and CuI (12 mg, 0.063 mmol, 0.13 equiv) was added in a 150 mL three-neck flask under a continuous N_2_ flow for 30 min. The mixture of *N*,*N*-dimethylformamide and triethylamine (8 mL/8 mL) was injected into the reaction system, which was further heated at 105 °C for 48 h under a N_2_ atmosphere and dark conditions. After cooling down to room temperature, the mixture was filtered and washed with fresh *N*, *N*-dimethylformamide, tetrahydrofuran, methanol, and HCl aqueous solution (0.1 M). The sample was dried at 120 °C under vacuum for 24 h to obtain PPOP-H (267 mg, 92% yield) as purple solids.

**Synthesis of PPOP-OH**


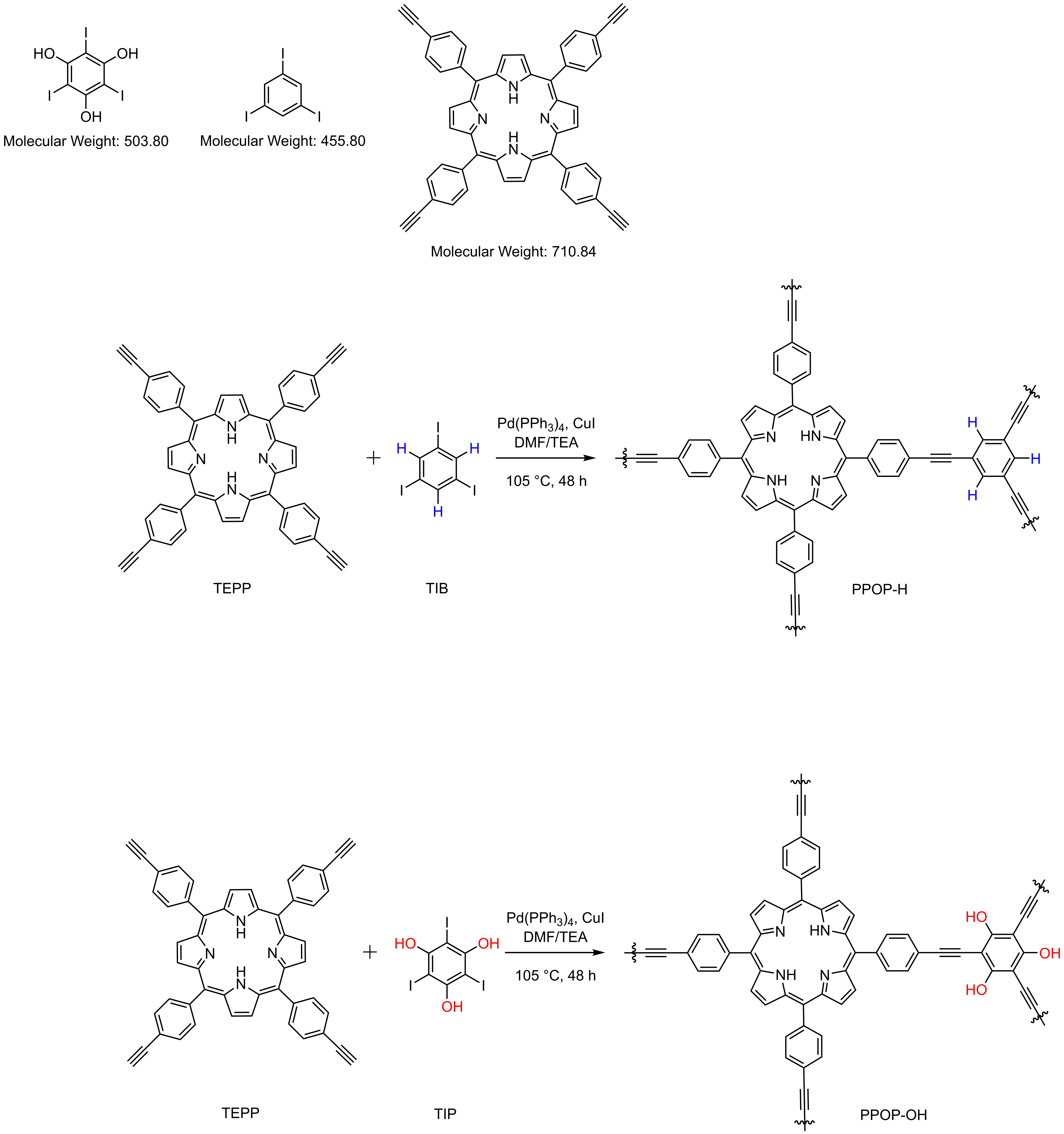


A mixture of TEPP (256 mg, 0.360 mmol, 0.750 equiv), TIP (242 mg, 0.480 mmol, 1.00 equiv), tetrakis(triphenylphosphine)palladium(0) (36 mg, 0.031 mmol, 0.065 equiv), CuI (12 mg, 0.063 mmol, 0.13 equiv) was added in a 150 mL three-neck flask under a continuous N_2_ flow for 30 min. The mixture of *N,N*-dimethylformamide and triethylamine (8 mL/8 mL) was injected into the system and heated at 105 °C for 48 h under a N_2_ atmosphere and dark conditions. The mixture was filtered after cooling down to room temperature, which was washed with fresh *N,N*-dimethylformamide, tetrahydrofuran, methanol, and HCl aqueous solution (0.1 M). The PPOP-OH was obtained after drying at 120 °C under vacuum for 24 h (292 mg, 93% yield) as purple solids.

**Fabrication of electrochemical aptasensors**

Au electrodes (AEs) were polished by Al powders (0.5 μm) and soaked in a mixed solution of H_2_O_2_/H_2_SO_4_ (v/v = 7/3). The phosphate buffer solution was prepared by dissolving Na_2_HPO_4_ (1.450 g), KH_2_PO_4_ (0.240 g), KCl (0.200 g), and NaCl (8.000 g) in distilled water (1.00 L), which was further adjusted to pH ~7.4 through the NaOH or HCl aqueous solutions (0.1 M). Both PPOPs were well dispersed in methanol (0.2 mg/mL). 10 μL of the suspensions were slowly dropped on the surface of AEs to obtain PPOPs modified electrodes (PPOP@AE), which were washed by distilled water for 5 min. The PPOP@AE was immersed in an aptamer solution at 10 ng/mL for 2 h and slowly washed by distilled water for 5 min to obtain the aptasensor (apt@PPOP@AE). The samples of apt@PPOP were prepared by the same soaking manner without using AEs.

**Electrochemical measurements**

Electrochemical impedance spectroscopy (EIS) was measured on CHI440C workstation (Shanghai Chenhua) with a three-electrode system, including AE as a working electrode, Ag/AgCl (saturated KCl) as a reference electrode, and Pt slide as a counter electrode. The mixed solution of KCl (0.100 M) and [Fe(CN)_6_]^3–/4–^ (0.500 mM) was used during the electrochemical measurements. EIS Nyquist plots were measured at an open circuit voltage over a frequency range of 100 kHz ~ 0.1 Hz at the amplitude of 5 mV.

**Determination of KANA in real samples**

Different real samples were added with KANA to study the sensing ability of the fabricated electrochemical aptasensors. Ultrapure water (18.0 mL) was added to pure milk (12.0 mL). The mixture was then treated with 10% trichloroacetic acid and chloroform (6.0 mL) under ultrasonic treatment for 20 min, which was centrifuged at 12,000 rpm for 15 min to collect the upper supernatant. The river sample was centrifugated at 12,000 rpm for 15 min. The upper supernatant was filtered through a membrane (0.22 μm). All samples were diluted 1000 times and further added different amounts of KANA.

1. **Supporting Data**


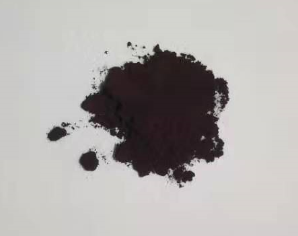

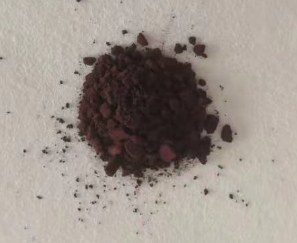


**Figure S1**. The optical photos of PPOP-OH (left) and PPOP-H (right).

**
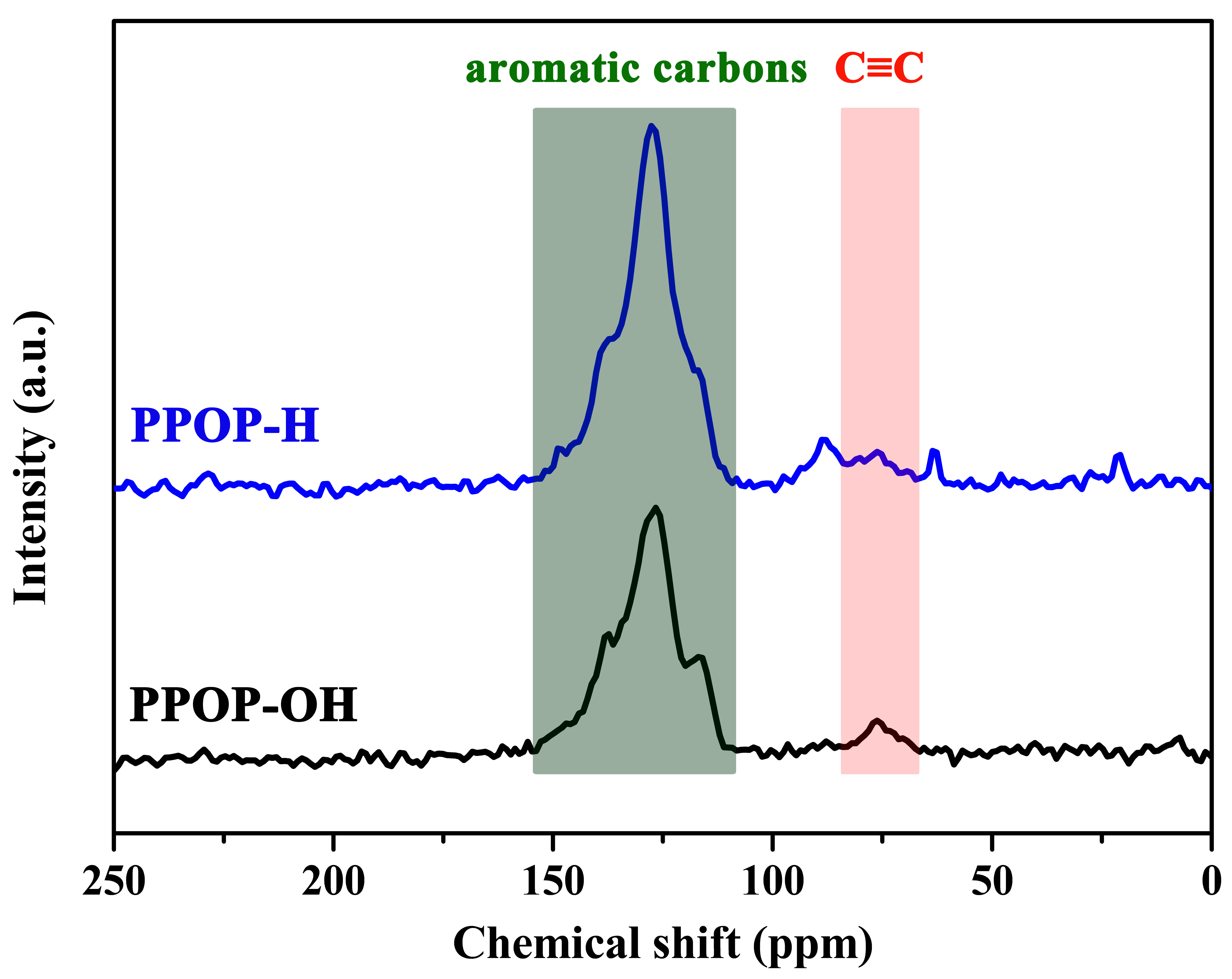
**

**Figure S2**. The solid-state ^13^C CP-MAS NMR spectra collected for PPOP-OH and PPOP-H.


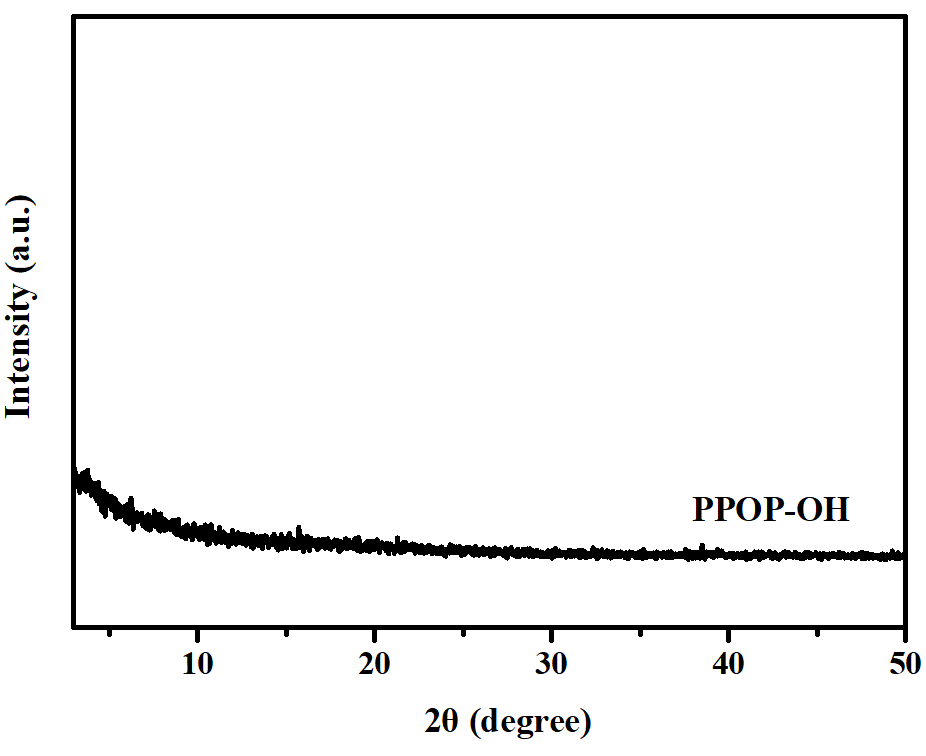


**Figure S3**. The PXRD patterns of PPOP-OH indicate its amorphous nature from the irreversible covalent bond formation process.


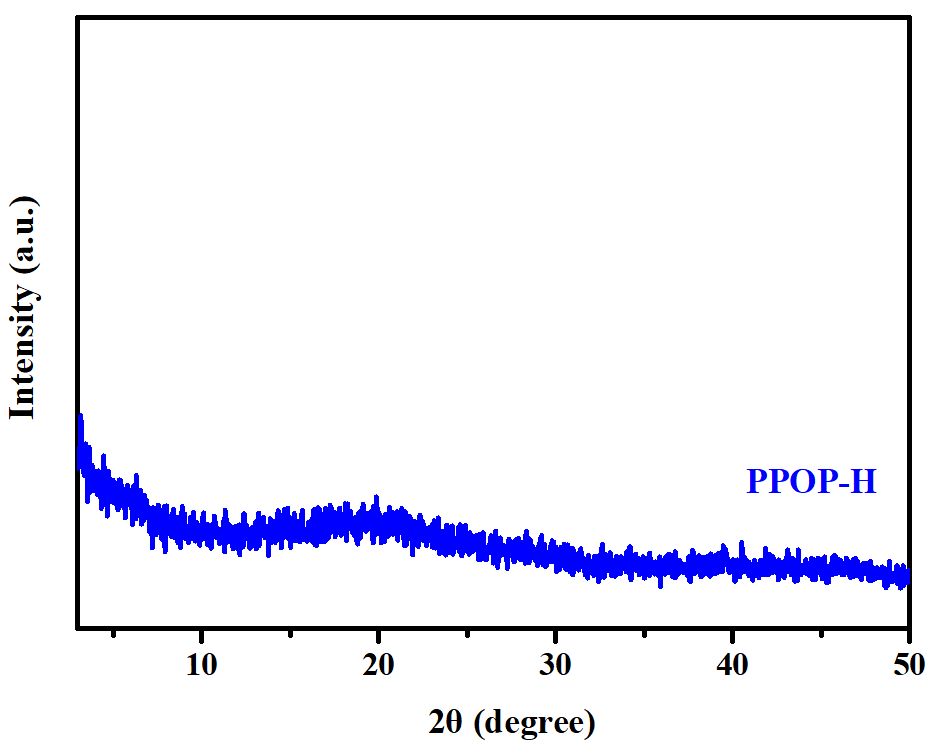


**Figure S4**. The PXRD pattern of PPOP-H indicate its amorphous nature from the irreversible covalent bond formation process.


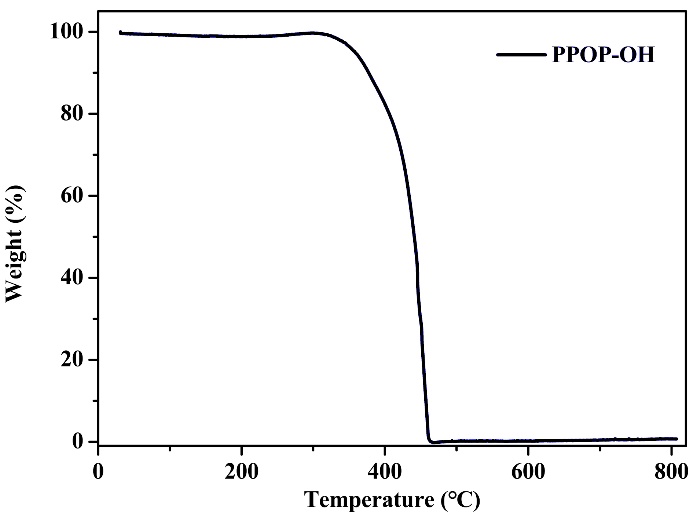


**Figure S5**. The thermogravimetric analysis plot of PPOP-OH reveals its great thermal stability up to 320 °C.


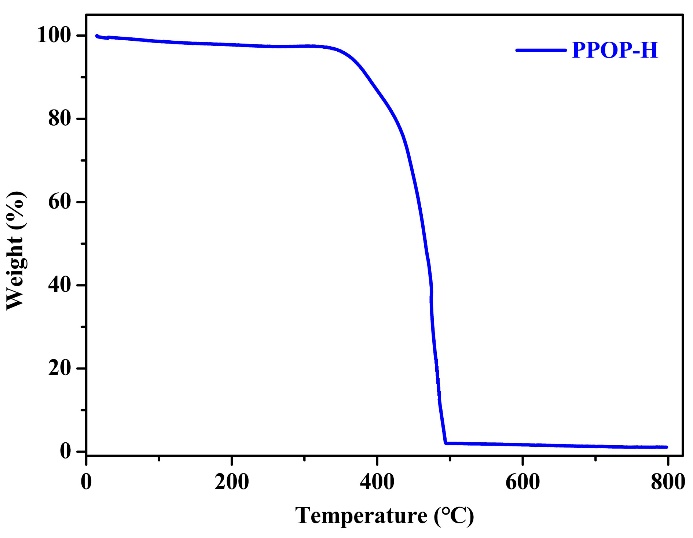


**Figure S6**. The thermogravimetric analysis plot of PPOP-H reveals its great thermal stability up to 320 °C.

**
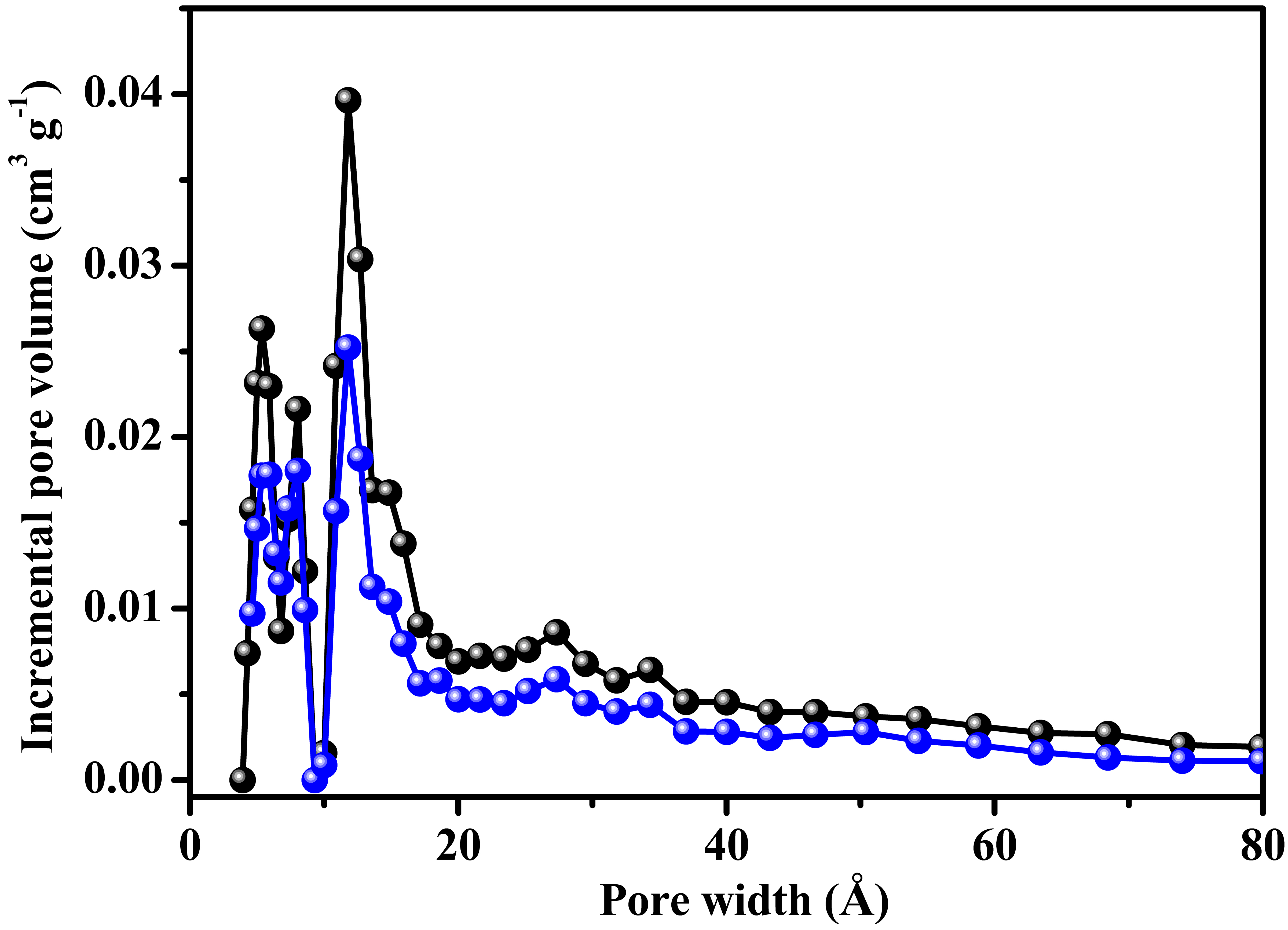
**

**Figure S7.** Pore size distributions of PPOP-H (black line) and PPOP-OH (blue line) are calculated nitrogen adsorption isotherms at 77 K.

**
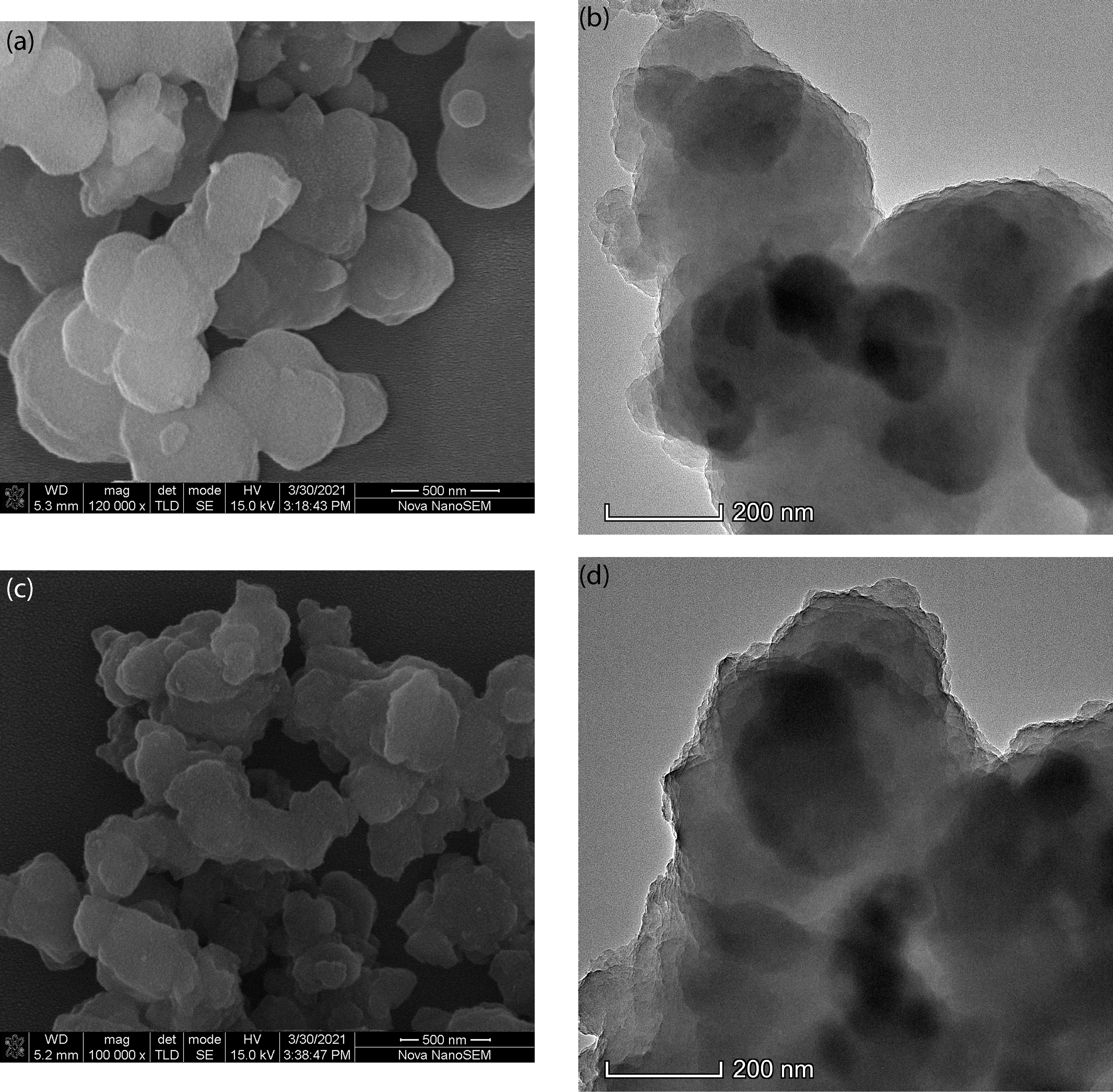
**

**Figure S8.** SEM and TEM images of PPOP-H (a and b) and PPOP-OH (c and d).


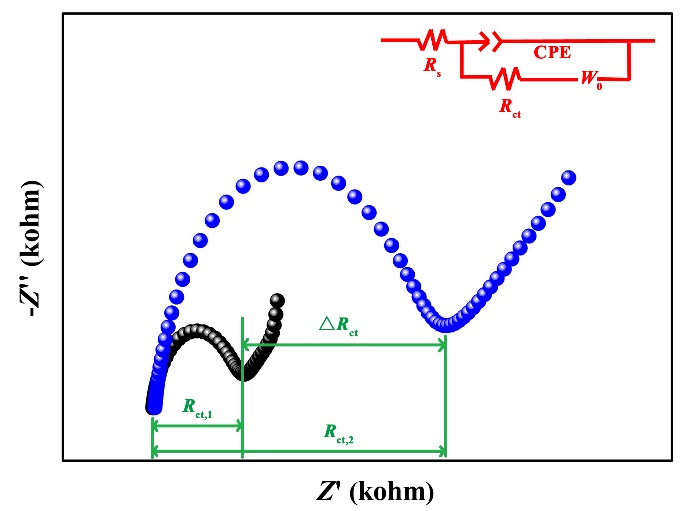


**Figure S9.** EIS Nyquist plots and equivalent circuit.


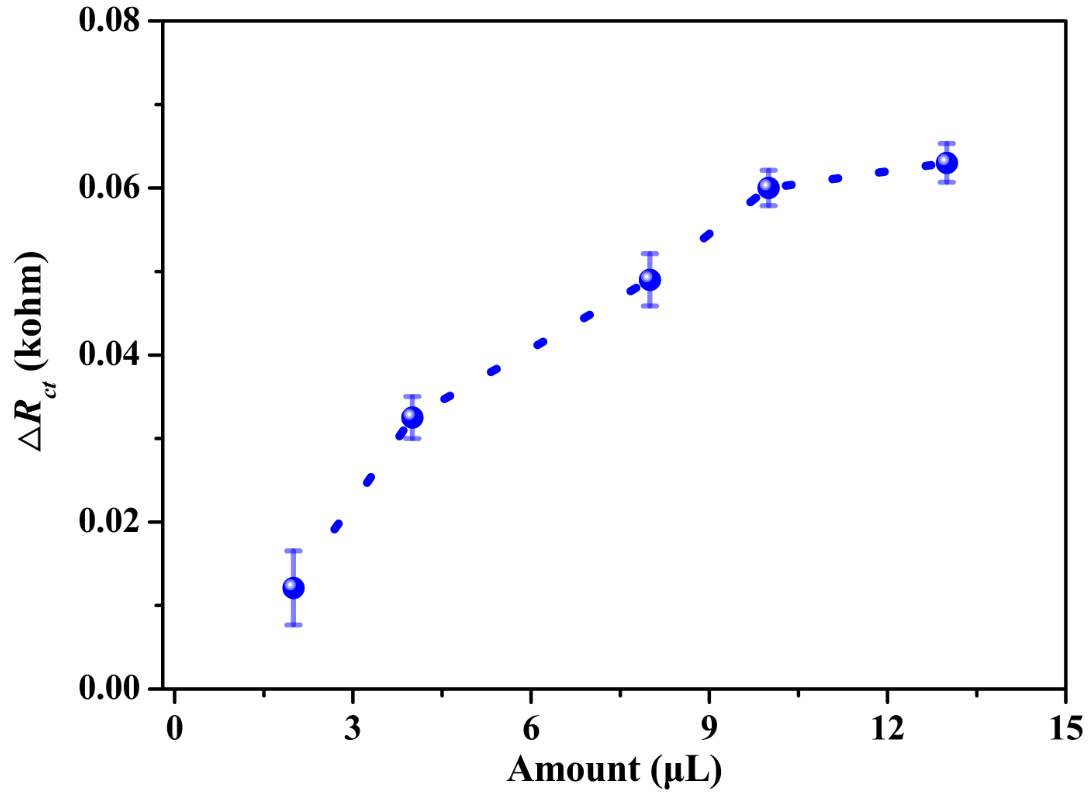


**Figure S10.** This set of experiments examines how different amounts of PPOP-OH added on AE influence the *R*_ct_ changes (Δ*R*_ct_ = *R*_ct,PPOP@AE_ – *R*_ct,AE_). The Δ*R*_ct_ value of PPOP-OH@AE increases to 0.06 kΩ after adding 10.0 μL of PPOP-OH (0.20 mg/mL in methanol), but no significant increase can be found with the further addition of PPOP-OH.


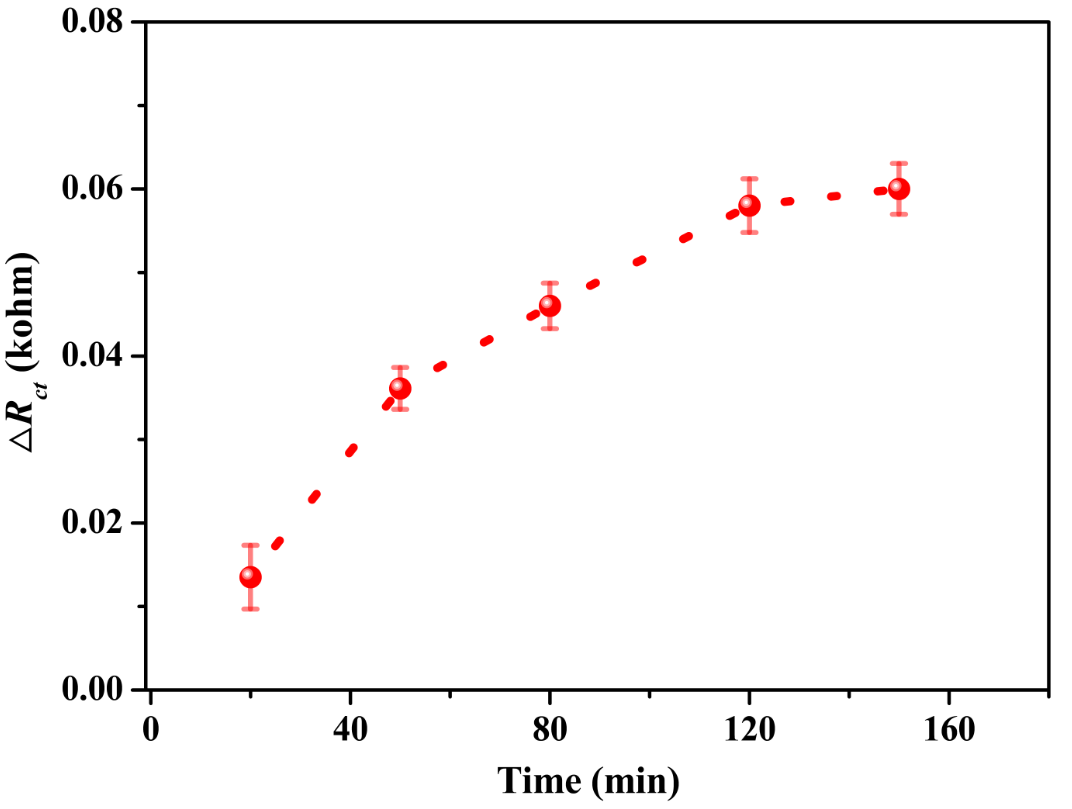


**Figure S11**. This set of experiments examines how the incubation time of PPOP-OH@AE in the aptamer solution influences the *R*_ct_ changes (Δ*R*_ct_ = *R*_ct,apt@PPOP@AE_ – *R*_ct,PPOP@AE_). The Δ*R*_ct_ value was significantly enhanced by extending its soaking time in the aptamer solution at 10 ng/mL for 2 h.


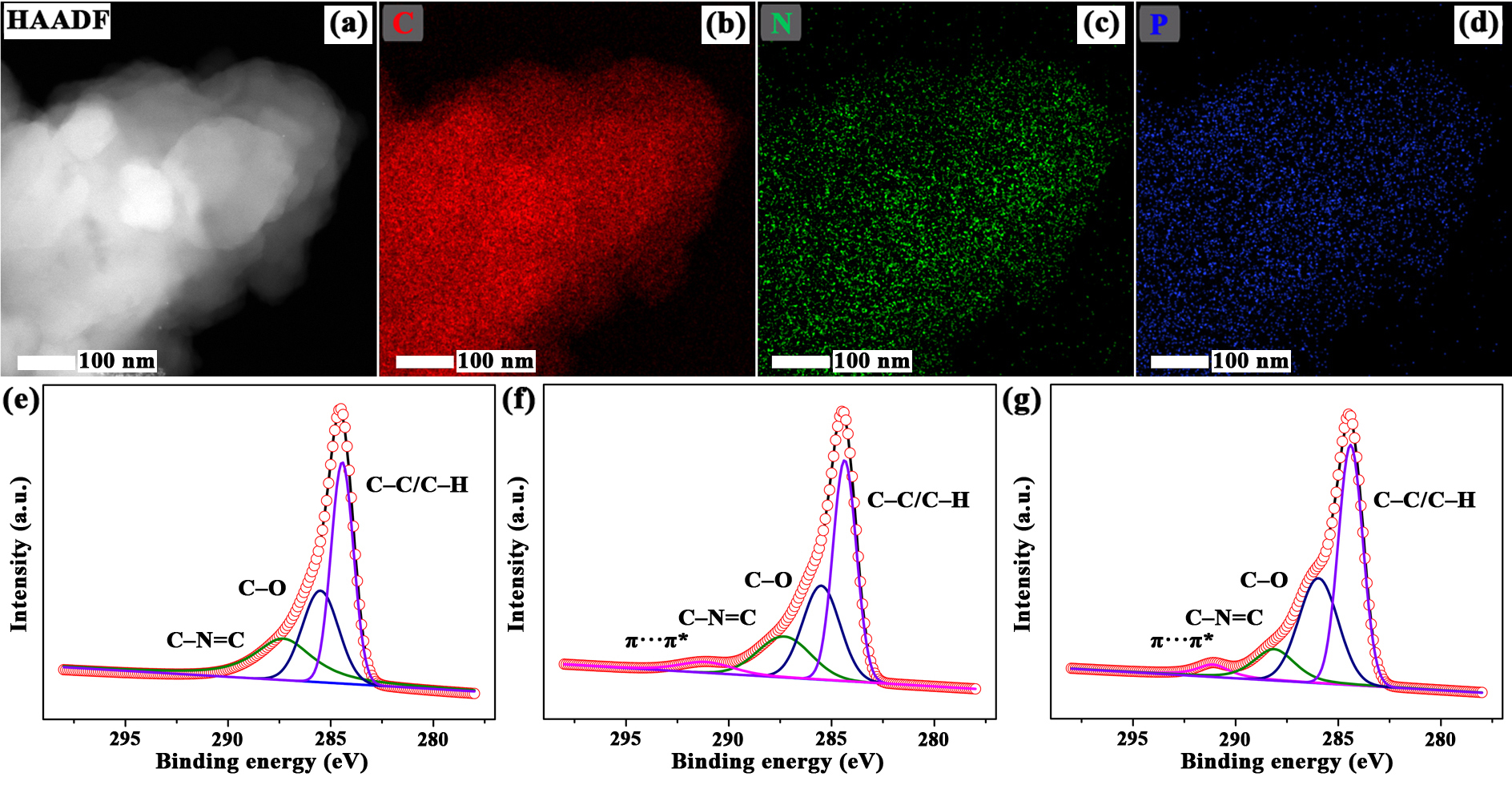


**Figure S12**. (a–d) The HAADF-STEM image and the elemental distributions of apt@PPOP-OH. High-resolution C1*s* XPS spectra of (e) PPOP-OH, (f) apt@PPOP-OH, and (g) KANA@apt@PPOP-OH.


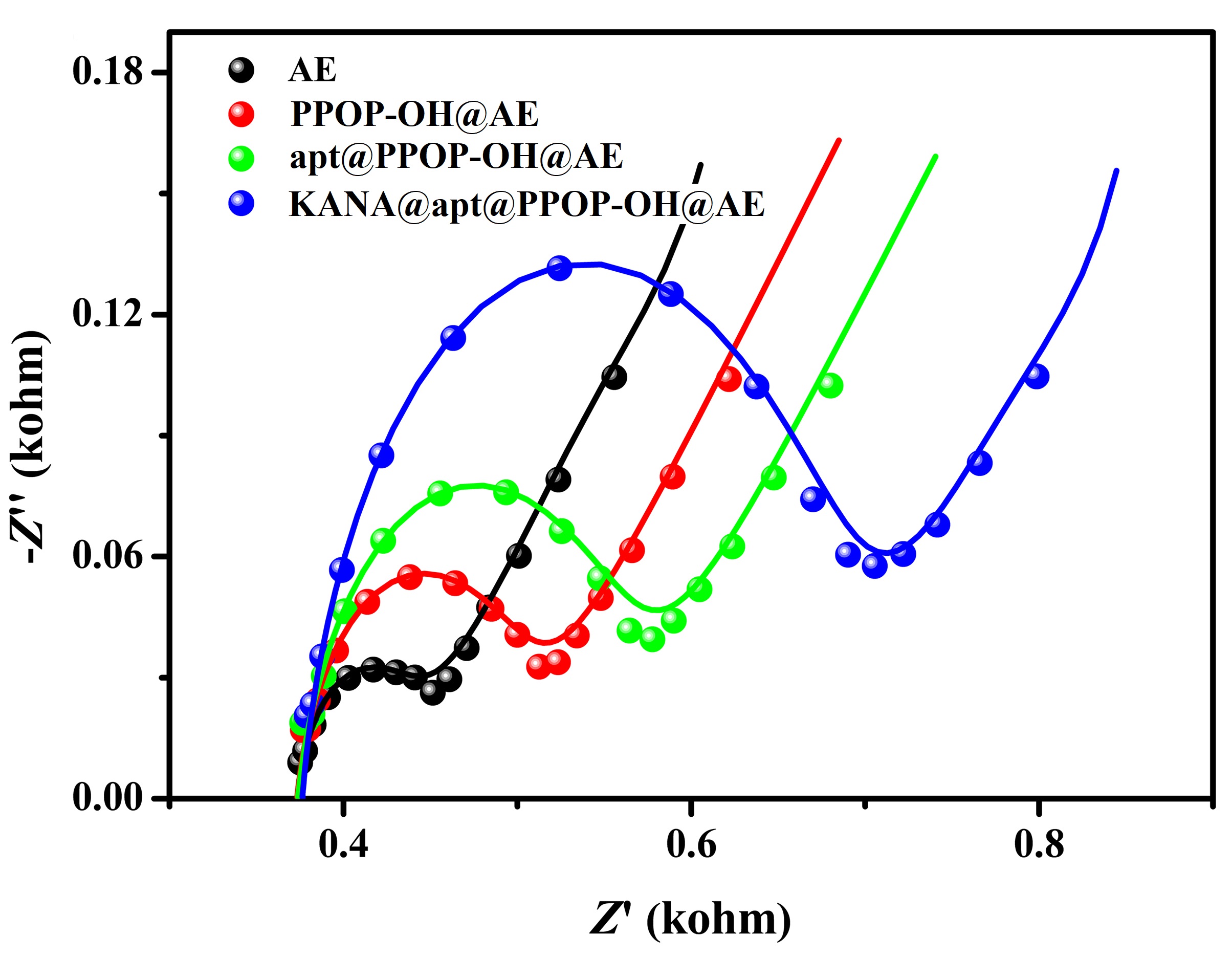


**Figure S13**. EIS Nyquist plots of AE, PPOP-OH@AE, apt@PPOP-OH@AE, and KANA@apt@PPOP-OH@AE.


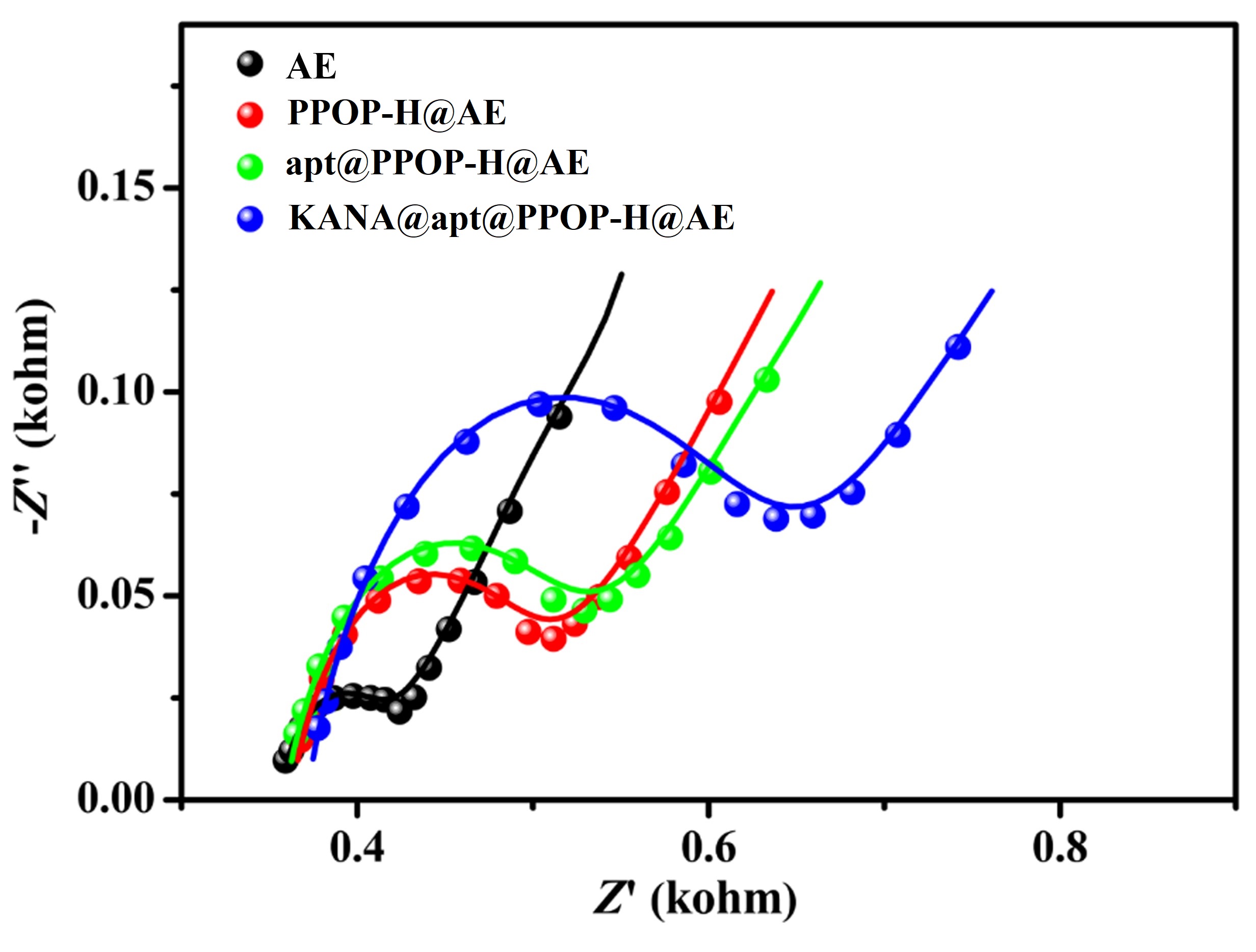


**Figure S14**. EIS Nyquist plots of AE, PPOP-H@AE, apt@PPOP-H@AE, and KANA@apt@PPOP-H@AE.


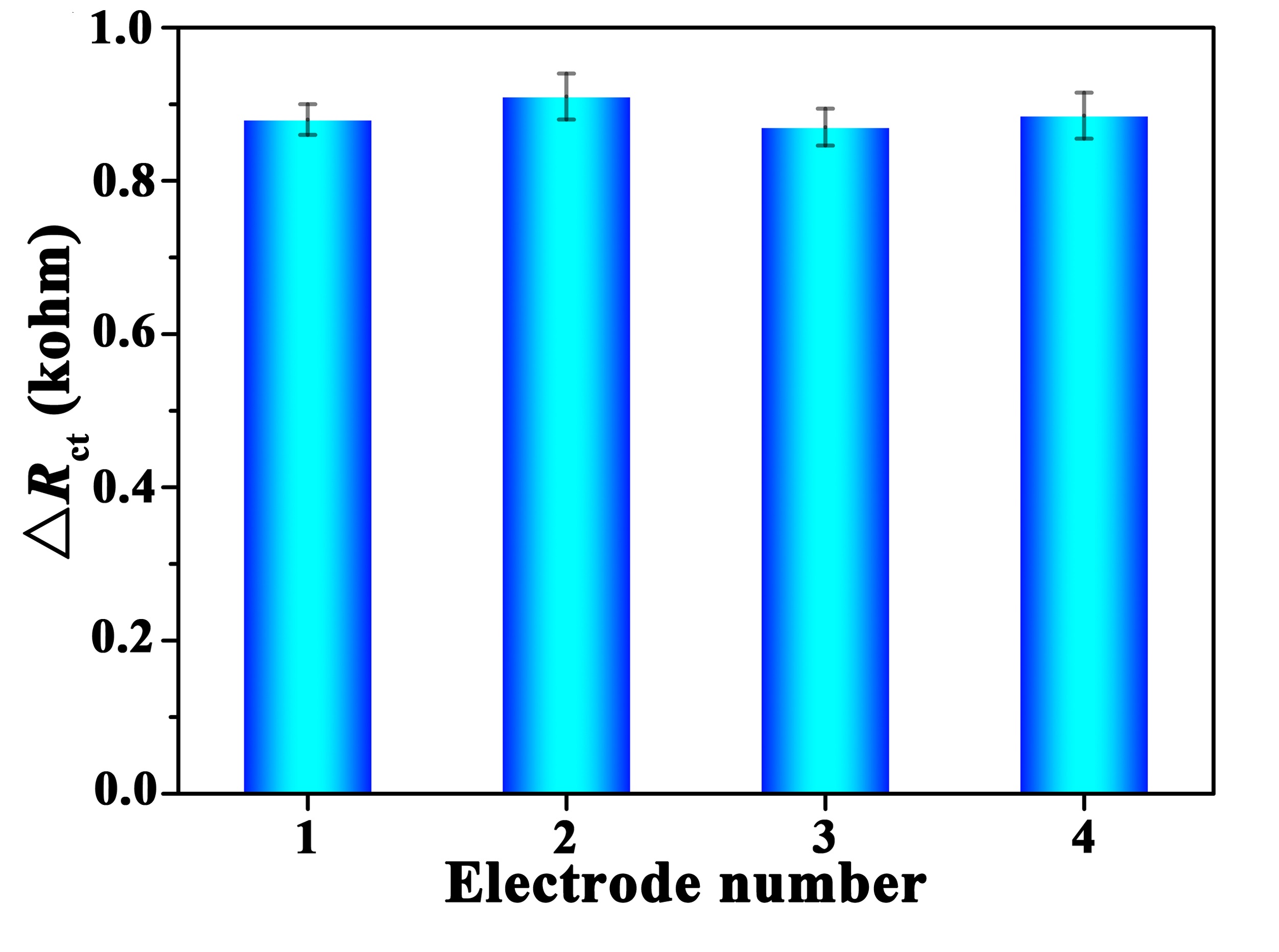


**Figure S15**. Reproducibility of the fabricated PPOP-OH-based electrochemical aptasensor for detecting KANA at 0.5 μg/L.


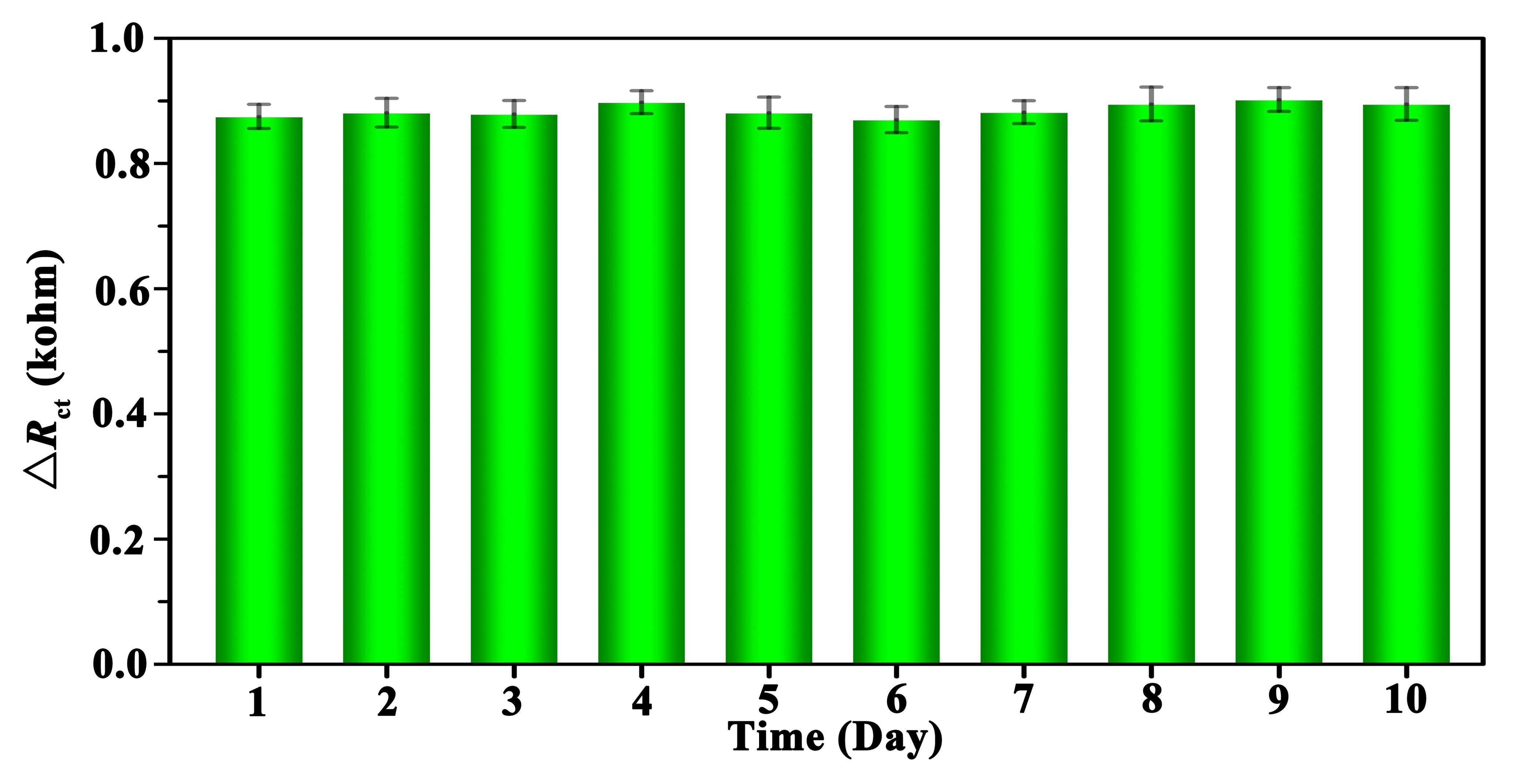


**Figure S16**. Stability of the fabricated PPOP-OH-based electrochemical aptasensor for detecting KANA over the time.

**Table S1.** Determination of KANA in different real samples.

| Sample | Added  concentration (ng/L) | Found  concentration (ng/L) | Recovery  (%) | RSD  (%) |
| --- | --- | --- | --- | --- |
| Milk | 0.100 | 0.097 | 97.0 | 3.92 |
|  | 1.00 | 1.03 | 103 | 2.87 |
|  | 100 | 104 | 104 | 4.15 |
| River | 0.100 | 0.103 | 103 | 3.76 |
|  | 1.00 | 0.990 | 99.0 | 2.61 |
|  | 100 | 105 | 105 | 4.39 |
